# ‘Flow’ Transcranial Direct Current Stimulation (tDCS) Device and On-Line Behaviour Therapy Training Software Used at Home for Perinatal and Maternal Loss Patients With Diagnosis of Depression

**DOI:** 10.1192/bjo.2025.10129

**Published:** 2025-06-20

**Authors:** Chris Griffiths, Ksenija da Silva, Anthony Dumi, Carol Abraham, Kirsty Harris

**Affiliations:** ^1^Northamptonshire Healthcare NHS Foundation Trust (NHFT), Northampton, United Kingdom; ^2^Coventry University, Coventry, United Kingdom

## Abstract

**Aims:** ‘Flow’ is a transcranial direct current stimulation (tDCS) treatment for depression that patients use at home. Meta-analyses of randomised sham-controlled trials (RCTs) show tDCS is safe, easy to use, and associated with significant improvements in depressive symptoms and high rates of clinical response and remission relative to placebo sham stimulation. Flow is BSI and CE-marked for treating depression in the UK, with NICE guidance for use in the NHS.

Flow incorporates an evidence backed healthy lifestyle behaviour training software app, and depression symptom tracking that enables users and their clinicians to monitor progress/symptoms. Training modules on: ‘Behaviour activation’, ‘Mindfulness’, ‘Exercise for your brain’, ‘An anti-depression diet’, and ‘Therapeutic sleep’.

In a first for the NHS, Northamptonshire Healthcare NHS Foundation Trust’s (NHFT) Specialist Perinatal Mental Health and Maternal Loss Psychology Service offered Flow to their patients with a diagnosis of depression and evaluated the feasibility and impact.

**Methods:** The patient self-administers Flow tDCS treatment, sessions last for 30 minutes, and are repeated 5 times weekly for 3 weeks, and after the initial 3-week period, patients self-administer 3 sessions per week for 3 weeks, and then as long as required. Outcome measure data collection from baseline to 6-week follow-up point. Self-report measures used were depression: Personal Health Questionnaire (PHQ-9); health related quality of life: EQ-5D-5L; and real-world functioning: Work and Social Adjustment Scale (WSAS). In-depth interviews were undertaken with 14 patients.

**Results:** There has been high level of adherence in the 25 participants to treatment protocol. There has been statistically significant improvements in depression symptoms (large effect size), real-world meaningful functioning, and health-related quality of life. Reliable improvement and remission rates for PHQ-9 were 64% and 52% respectively. In in-depth interviews most participants described a positive impact on depressive symptoms, sleep, social life, and functioning.

**Conclusion:** Flow has been successfully integrated into Perinatal Mental Health and Maternal Loss Psychology Service depression treatment offer. It is important to offer NHS patients an evidence-backed alternative to existing depression treatments (antidepressant medication and talking therapies), many patients stop antidepressants when they become pregnant, and many do not tolerate antidepressants side effects or wish to try due to side effects and withdrawal issues. Findings provide support for the approach of delivering both tDCS and on-line wellbeing behaviour therapy training to patients with experience of depression.

